# Expression of a Codon-Optimized *dsdA* Gene in Tobacco Plastids and Rice Nucleus Confers D-Serine Tolerance

**DOI:** 10.3389/fpls.2016.00640

**Published:** 2016-05-12

**Authors:** Yanmei Li, Rui Wang, Zongliang Hu, Hongcai Li, Shizhan Lu, Juanjuan Zhang, Yongjun Lin, Fei Zhou

**Affiliations:** National Key Laboratory of Crop Genetic Improvement and National Centre of Plant Gene Research, Huazhong Agricultural UniversityWuhan, China

**Keywords:** *Nicotiana tabacum*, *Oryza sativa*, selectable marker, D-serine, D-serine ammonia lyase, plastid transformation, nuclear transformation

## Abstract

D-serine is toxic to plants. D-serine ammonia lyase, which is encoded by the *dsdA* gene, can attenuate this toxicity with high specificity. In the present study, we explored the function of codon-optimized *dsdA* with tobacco plastids and rice nuclear transformation system. It was shown that *dsdA* gene was site-specifically integrated into the tobacco plastid genome and displayed a high level of expression. Genetic analysis of the progenies showed that *dsdA* gene is maternally inherited and confers sufficient D-serine resistance in tobacco. The effective screening concentrations of D-serine for seed germination, callus regeneration and foliar spray were 10, 30, and 75 mM, respectively. In addition, calluses from homozygous transgenic rice lines also showed significant tolerance to D-serine (up to 75 mM). Our study proves the feasibility of using *dsdA* gene as a selectable marker in both plastid and nuclear transformation systems.

## Introduction

As the essential organelle for photosynthesis, chloroplast has attracted great research interests. Compared with nuclear transformation, plastid transformation has its unique advantages (Daniell and Dhingra, 2002; Bock, 2014). Foreign genes can be site-specifically integrated into the plastid genome by homologous recombination mechanisms, which could eliminate the position effects and gene silencing (Dhingra et al., 2004; Ruhlman et al., 2010). Plastid genome is characterized by high copy number, which provides a platform for the high-level expression of exogenous genes (Lee et al., 2003; Koya et al., 2005; Oey et al., 2009a; Li et al., 2013). Furthermore, their maternal inheritance property greatly reduces cross-pollination and thus decreases the risk of biosecurity (Ruf et al., 2007; Schneider et al., 2015). In addition, polycistronic expression in plastid can organize multiple transgenes together for effective expression (Daniell and Dhingra, 2002; Zhou et al., 2007; Lu et al., 2013). Over the past several decades, plastid transformation has been successfully applied in dicotyledonous plants (Ruf et al., 2001; Craig et al., 2008; Scotti et al., 2009; Soria-Guerra et al., 2009), including the application of bioreactors (Kolotilin et al., 2012; Clarke et al., 2013; Gorantala et al., 2014; Hassan et al., 2014) and the improvement of agronomic traits in different plant species, such as resistance to herbicides (Ye et al., 2001; Kang et al., 2003) and insects (Hou et al., 2003; Liu et al., 2008; Zhang et al., 2015a), tolerance to salt (Kumar et al., 2004), and drought (Lee et al., 2003).

Despite the wide application of plastid transformation in dicotyledons, relatively fewer studies of plastid transformation have been reported in monocotyledons, especially in rice (Khan and Maliga, 1999; Lee et al., 2006; Li, 2013), which is one of the most important food crops in the world. Hence, the establishment of plastid transformation in rice will greatly help the sustainable development of agriculture. Inefficient transformant selection is considered the main obstacle to the implementation of plastid transformation in rice. The most commonly used genes for transformant selection in plastid transformation are antibiotic resistance genes, such as *aadA* (encoding aminoglycoside 3′—adenylyltransferase), *nptII* (encoding neomycin phosphotransferase), and *CAT* (encoding chloramphenicol acetyltransferase), which confer resistance to spectinomycin, kanamycin and chloramphenicol, respectively (Svab et al., 1990b; Carrer et al., 1993; Li et al., 2011). Spectinomycin is widely used as a screening substrate in plastid transformation of dicotyledonous plants due to its unique high specificity and low side-effect to plant cells. However, cereals are insensitive to spectinomycin and streptomycin (Li, 2013). Thus, these antibiotics can not be applied in the plastid transformation of crop plants, such as rice. Therefore, the discovery of more candidate genes with screening potential for transformant selection is very critical to the development of plastid transformation in rice. Moreover, the use of antibiotic resistance markers in transgenic breeding might be questioned by consumers due to the potential risk for environment and food safety. Hence, the application of non-antibiotic marker genes in the selection of transformants has become a hot spot in research. In recent years, the metabolic pathway genes, such as *ASA2* (Barone et al., 2009) and *dao* (Gisby et al., 2012), have been successfully used in tobacco plastid transformation system. Plants have been shown to be sensitive to D-serine (Valdovinos and Muir, 1965). A putative cause of D-amino acid toxicity to plants is the competitive binding to tRNA (Erikson et al., 2004). The *dsdA* from *Escherichia coli*, which encodes D-serine ammonia lyase, can attenuate the toxicity of D-serine to plants by catalyzing the deamination of the D-serine to pyruvate, ammonium and water (Federiuk et al., 1983). The *dsdA* gene has been reported to be successfully used as a selectable marker in *Arabidopsis thaliana* nuclear transformation system, and there is no direct interference with endogenous amino acid metabolism (Erikson et al., 2005). Moreover, recently D-serine has been demonstrated to act as a neuromodulator in humans (De Miranda et al., 2002), and can be added to established antipsychotic drugs for treatment of schizophrenic patients (Tsai et al., 1998). Although it has been shown that excessive D-serine is nephrotoxic (Krug et al., 2007), D-amino acid oxidases in animals can detoxify D-amino acids accumulated *in vivo* (D'Aniello et al., 1993). Besides, D-serine is considered to be relatively nontoxic to animals and microbes (Friedman, 1999; Krishnamoorthy et al., 2012; Kuru et al., 2012), which might relieve the consumers' concern about the safety of transformants. Therefore, it is necessary to explore the feasibility of using *dsdA* gene for selecting and screening transformants and to investigate the tolerance capacity against D-serine in different systems (such as monocots and dicots).

In this study, the construct carrying *dsdA* gene was introduced into the tobacco plastome by biolistic method, resulting in homoplastomic transformants with sufficient resistance to D-serine. Moreover, the homozygous *dsdA* transgenic rice with single-copy insertion from nuclear transformation was identified, which also showed increased resistance to D-serine. Our study not only provides useful information about the usage of D-serine in tobacco and rice but also proves the feasibility of using *dsdA* gene as a selectable marker in both plastid and nuclear transformation systems. Furthermore, it is proposed that *dsdA* gene can be used as an effective candidate gene in the plastid transformation of monocotyledons.

## Materials and methods

### Plant materials and growth conditions

Tobacco plants (*Nicotiana tabacum* cv. Petit Havana) were grown under sterile conditions on agar-solidified MS medium supplemented with 30 g/L sucrose (Svab and Maliga, 1993). Genetically modified plants were selected, propagated, and rooted on the same medium. For sampling and seed production, plants were transferred to soil and grown under standard greenhouse conditions (25°C, 16 h light/8 h darkness; light intensity, 50–200 μE m^−2^ s^−1^).

### Construction of plastid transformation vector

The *dsdA* plastid transformation vector was constructed based on the previously described vector pZF75 (Zhou et al., 2007). The *dsdA* sequence from NCBI (Gene ID: 946837) was codon-optimized for expression in the plastids and chemically synthesized under the premise of no restriction sites harbored in the gene (Genscript, China). The coding region of *dsdA* was then PCR-amplified using primers *dsdA*-F (5′-GTCGAC*GGGAGGGATTTC C*ATGGAAAATGCTAAAATGA-3′, the underlined cases are *Sal*I site.) and *dsdA*-R (5′-CTCGAGTTATCTTCC TTTAGCTAAATATTG - 3′, the underlined cases are *Xho*I site.). With these primer sequences, the *rbcL* leader sequence containing the Shine–Dalgarno sequence (in italics in the *dsdA*-F sequence) and the restriction sites *Sal*I and *Xho*I were introduced into the PCR product. The resulting amplification product was digested with *Sal*I and *Xho*I, and ligated into the corresponding sites by replacing the *yfp* gene in plasmid pZF75, generating the final plastid transformation vector pZF75-dsdA.

### Plastid transformation and selection of transplastomic lines

Plastid transformation was carried out using biolistic protocol (Svab et al., 1990a), 4-week-old leaves harvested from sterile tobacco plants were bombarded with plasmid DNA-coated 0.6 μm gold particles by using the PDS1000He biolistic gun with the Hepta Adapter setup (Bio-Rad, http://www.bio-rad.com/). Primary spectinomycin-resistant shoots were selected on the regeneration medium containing 500 mg/L spectinomycin. In order to prevent the selection of spontaneous spectinomycin mutants, resistant shoots were also tested on a streptomycin/spectinomycin double selection medium (500 mg/L each; Bock, 2001; Svab and Maliga, 1993). Transplastomic lines were selected for two rounds by regeneration on antibiotic selection medium to obtain homoplastomic plants after particle bombardment (Zhou et al., 2007).

### Isolation of nucleic acids and gel blot analyses

Total plant genomic DNA was isolated from fresh leaf tissue using the Cetyltrimethyl Ammonium Bromide (CTAB) method (Doyle and Doyle, 1990). For Southern blot analysis, DNA samples (8 μg total cellular DNA) were digested with *Bgl*II and separated by gel electrophoresis in 0.8% agarose gels, and then transferred onto nylon membrane. A 550-bp digoxigenin (DIG) labeled probe was generated by amplification of a portion of the *psaB* coding region with primers (7244: 5′-CCCAAGGGGCGGGAACTGC-3′ and 7247:5′-CCCAGAAAGAGGCTGGCCC -3′). This probe was used to verify plastid transformation and assess the homoplastomic state. Hybridization was performed according to the procedure described by manufacturer's protocols (Roche, Mannheim, Germany). RNA was extracted using the TriZol reagent according to a previous work (Wang et al., 2015). Total cellular RNA samples (10 and 15 μg total RNA) were electrophoresed in formaldehyde containing 1% agarose gels and transferred onto nylon membrane for Northern blot analysis. The *dsdA* probe labeled with DIG was amplified from the recombinant PZF75-dsdA vector using gene-specific primers NdsdA-F (5′-GGAGTTGCTGTTGAAGAAGGA-3′) and NdsdA-R (5′-TTGAGGTCCAGCC ATTCCA-3′). RNA blots were hybridized under the same conditions as for Southern blot analysis at 52°C.

### Homoplasmy and maternal inheritance analysis

To confirm homoplasmy and maternal transgene inheritance, transplastomic plants grown to maturity under greenhouse conditions were self-pollinated and reciprocally crossed to wild-type plants. Seeds were harvested and germination assay was performed on MS medium with kanamycin (Kan; 400 mg l^−1^), medium with spectinomycin (Spec; 500 mg l^−1^), and medium with both antibiotics (Spec + Kan).

### D-serine tolerance assay of transplastomic tobacco plants

The seeds from transplastomic tobacco plants after surface-sterilization were germinated on MS medium (Murashige and Skoog, 1962) with or without D-serine (RRID: *Alfa Aesar: A11353*) for growth assay. The aerial parts of young plants were sampled for fresh weight measurements. For shoot regeneration assay, leaves from aseptic plants were placed on RMOP regeneration medium (Svab and Maliga, 1993) with or without D-serine. In addition, D-serine solution (75 mM) containing Tween 20 (0.2%, v/v) was used to spray the leaves of tobacco lines grown in soil.

### Selection of homozygous *dsdA* transgenic rice with single copy insertion

In this study, *dsdA* transgenic plants in T_0_ generation generated previously (Zhang et al., 2015b) were used to test the function of *dsdA* in Zhonghua 11 (ZH11) (*Oryza sativa* L. ssp. *japonica*). Total plant genomic DNA was isolated from fresh leaf tissue by using the CTAB method (Murray and Thompson, 1980). For Southern blot analysis, DNA samples (8 μg total cellular DNA) were digested with *Hind*III and separated by gel electrophoresis in 0.8% agarose gels, and then transferred onto nylon membrane according to the manufacturer's protocols. The DIG-labeled probe was prepared from a PCR-amplified fragment of hygromycin phosphotransferase (hpt) gene (Hyg-F: 5′-gatgttggcgacctcgtatt-3′ and Hyg-R: 5′-gtgtcacgttgcaagacctg-3′). For homozygous identification, 60 mature seeds of T_1_ transgenic plant were harvested from individual plants. The seed germination assay was conducted by the procedure previously described (Chen et al., 2005).

### Analysis of *dsdA* expression in homozygous transgenic lines with single-copy insertion

Expression levels of *dsdA* gene in transgenic lines were measured by quantitative real-time PCR (qRT-PCR). Total RNA from homozygous *dsdA* transgenic plants with single-copy insertion was extracted using the standard Trizol method (Transgen, China). Two micrograms of total RNA was used for reverse transcription according to the manufacturer's instructions (Invitrogen, USA). qRT-PCR was performed following the procedure described previously (Wang et al., 2015). The primers of the endogenous *actin* gene (LOC_Os03g50885) were qRTactin-F: (5′-TGGCATCTCTCAGCACATTCC-3′) and qRTactin-R (5′-TGCACAATGGATGGGCCAGA-3′), and the primers of *dsdA* were qRTdsdA-F: 5′-TCATTGCTTCTTTGCTGAACCTAC-3′ and qRTdsdA-R: 5′- CCTTCTTCTTGAGCTAACCATCCT-3′. Relative expression levels were determined using 2^−ΔΔCT^ method (Livak and Schmittgen, 2001).

### D-serine tolerance assay of *dsdA* transgenic rice

Seeds from homozygous *dsdA* transgenic lines with single-copy insertion and the wild-type (Zhonghua11, a *japonica* rice) were used for callus induction as previously described (Hiei et al., 1994). The calluses were induced for 35 days and then cultured on the subculture medium for 15 days before the test. The average diameter of the callus in the test was 0.25 cm. Then the calluses from transgenic line and wild-type were respectively placed on selective medium containing 75 mM D-serine for 28 days. To test the sensitivity of various rice varieties to D-serine, seeds from the *japonica* rice varieties [Zhonghua11, Dongjin, Nipponbare and the transgenic lines (4, 14 and 19)] and the *indica* rice varieties (Minghui63 and Zhenshan 97) were surface-sterilized and germinated on fresh 1/2 × MS medium containing 0, 5, 10, 15, and 20 mM D-serine for 9 days.

## Results

### Construction of *dsdA* expression vector for plastid transformation

**Figure 1:**
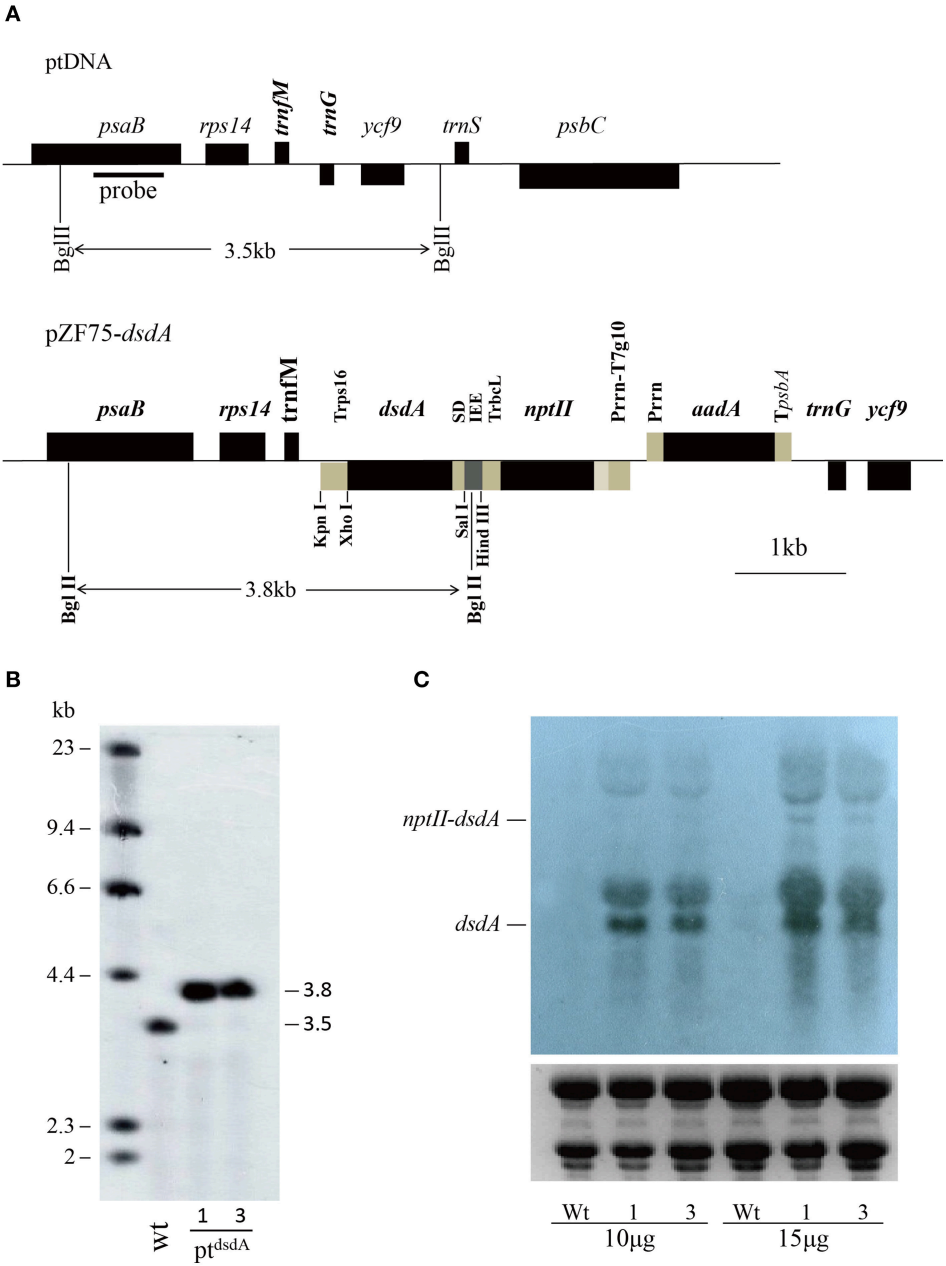
**Introduction of *dsdA* gene into the tobacco plastid genome**. **(A)** Physical map of the targeting region in the tobacco plastid genome of the wild-type (ptDNA) and the transplastomic lines (pZF75-*dsdA*). Map positions are shown as: P*rrn*: rRNA operon promoter; P*rrn*-T7g10: rRNA operon promoter fused to the leader sequence from bacteriophage T7 gene 10; IEE: Intercistronic Expression Element; SD: Shine-Dalgarno sequence; T*psbA*: 3′ UTR from the *psbA* gene; T*rbcL*: 3′ UTR from the *rbcL* gene; T*rps16*: 3′ UTR from the *rps16* gene. Genes above the lines are transcribed from left to right; genes below the lines are transcribed from right to left. The cleavage sites marked by thin lines were used for cloning. The operons of two transgenes (*nptII* and *dsdA*) were targeted to the intergenic region between the *trnfM* and *trnG* genes. The psaB gene was used as a probe for Southern blot analysis and is marked by a bold line; the wild-type (3.5 kb) and transplastomic (3.8 kb) fragments generated by the restriction enzyme *Bgl*II are marked by thin lines. **(B)** The DNA samples were digested with *Bgl*II and separated on a 0.8% agarose gel and then hybridized with a DIG-labeled psaB probe for Southern blot analysis to confirm proper integration and homoplasmy of the plastid *dsdA* gene. Digestion with *Bgl*II produces fragments of approximately 3.5 kb in the wild-type and 3.8 kb in two transplastomic lines, which correspond to the expected sizes of the three integrated transgenes. **(C)** Analysis of *dsdA* accumulation in transplastomic plants. Samples of total RNA were fractionated by denaturing gel electrophoresis and then transferred to nylon membranes with DIG-labeled *dsdA* probe for Northern blot analysis.

To explore the potential of D-serine to be used for selecting and screening transplastomic plants, we investigated the function of D-serine tolerance gene *dsdA*, which encodes D-serine ammonia lyase in transplastomic lines. Codon usage adaptation can result in a substantial increase of protein accumulation (Tregoning et al., 2003**;** Maliga, 2004). The examination of the codon usage in the native *dsdA* genomic sequences of *E. coli str. K12 substr. MG1655* (Gene ID: 946837) indicated that it differed substantially from that in the plastid genome of higher plants. Therefore, we re-synthesized the *dsdA* sequence and replaced the codons by the most preferred codons in the plastid. The previously described pZF75-based plastid transformation vector (Zhou et al., 2007) was employed, in which two transgenes were linked together in an operon: the kanamycin resistance gene *nptII* and the D-serine tolerance gene *dsdA* (Figure 1A). The polycistronic operon expression cassette was driven by the tobacco plastid 16S ribosomal RNA operon promoter (P*rrn*) fused to the phage T7-derived gene 10 leader sequence (T7g10, Kuroda and Maliga, 2001). To ensure effective processing of the polycistronic operon, previously identified intercistronic expression element (IEE) was used to link the first (*nptII*) and second (*dsdA*) cistrons. Terminator (T*rbcL*) derived from the plastid *rbcL* gene was used to stabilize the first monocistronic transcript (*nptII*) after processing. The second cistron (*dsdA*) was fused with a Shine–Dalgarno sequence to mediate its translation initiation, which was terminated by the *rps16* terminator (Staub and Maliga, 1994; Wurbs et al., 2007). The final transformation vector (Figure 1A) was designed for incorporating the *nptII-dsdA* operon together with the selectable marker gene *aadA* into the plastid genome between the *trnfM* and *trnG* genes (Ruf et al., 2001).

### Selection of transplastomic tobacco lines by southern blot analysis

The construct was introduced into the tobacco plastid genome by particle gun-mediated transformation (Svab and Maliga, 1993). Transplastomic lines were selected on regeneration medium with spectinomycin, and homoplasmy was ensured by one additional round of selection and regeneration on the medium with both spectinomycin and streptomycin.

To confirm plastid transformation and assess the homoplasmy of the transplastomic lines, total leaf DNA from two plastid transformants isolated from independent transformation events was analyzed by Southern blot analysis. A 550-bp DNA fragment corresponding to a part of the *psaB* coding region was used as a probe to distinguish homoplastomic and heterogeneous lines. The DIG-labeled *psaB* probe was hybridized to *Bgl*II digested genomic DNA. The *Bgl*II fragments with expected sizes of 3.5 and 3.8 kb were observed in the wild-type (Figure 1B) and the pt^*dsdA*^ transplastomic lines (Figure 1B), respectively. The absence of 3.5 kb fragment in pt^*dsdA*^ confirmed the homoplasmy of the transplastomic lines.

### High expression of *dsdA* gene in transplastomic tobacco plants

**Figure 2:**
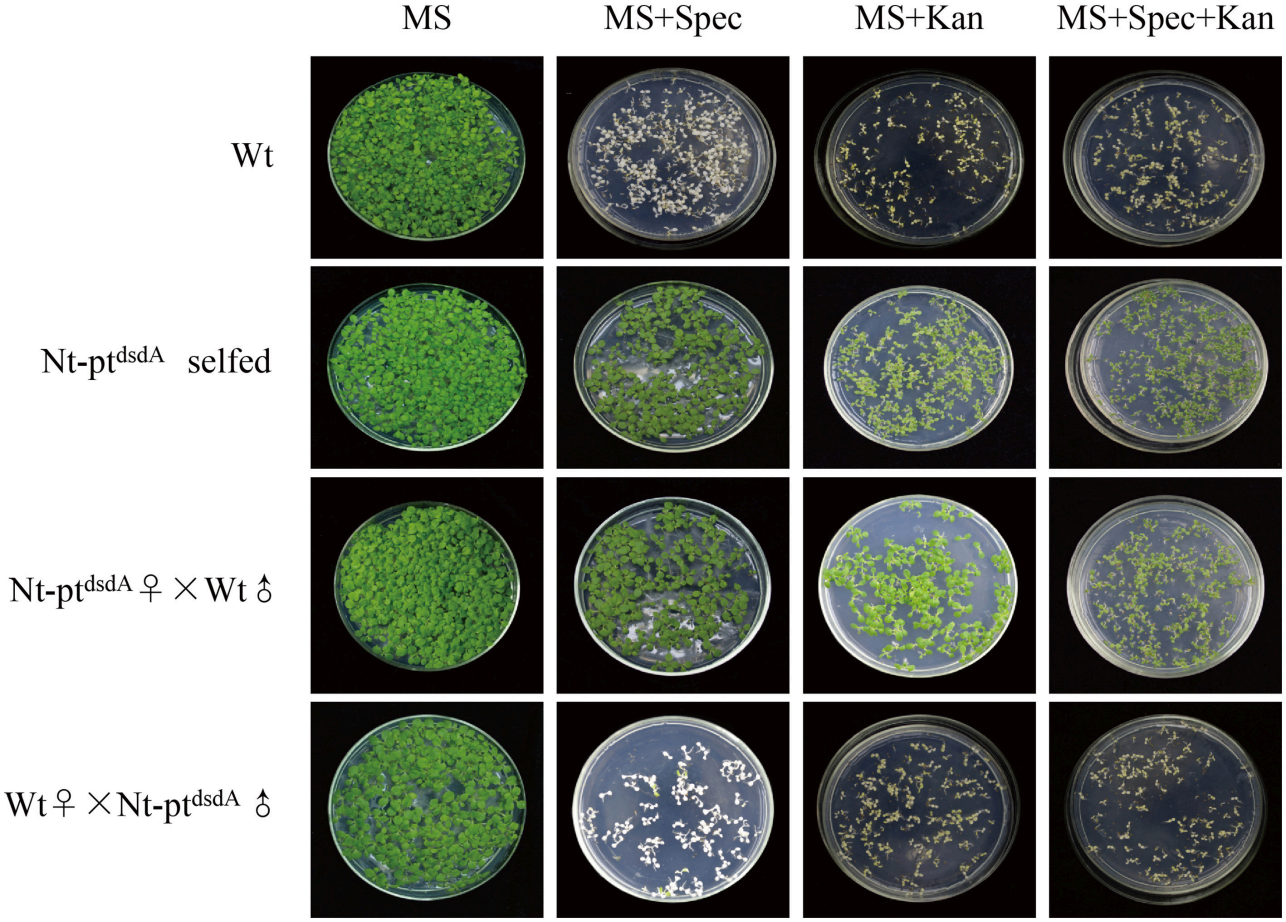
**Seed assays to confirm the homoplasmy of transplastomic plants and maternal inheritance**. Seeds from the wild-type, the transplastomic plants, and the reciprocal cross between them were germinated on antibiotic-free medium, medium with kanamycin (Kan; 400 mg l^−1^), medium with spectinomycin (Spec; 500 mg l^−1^), and medium with both antibiotics (Spec + Kan). The seeds of T1 generation showed no segregation, confirming the homoplasmy and maternal inheritance of the transplastomic lines.

After successful generation of homoplastomic plants, Northern blot analysis was conducted to detect the expression of *dsdA* gene in two independent transplastomic lines. There was no transcript band in the wild-type line, while identical transcript bands were detected in the two transplastomic lines (Figure 1C): a 1.3-kb hybridizing band corresponding to the monocistronic *dsdA* transcript and a weak and larger hybridizing band corresponding to the di-cistronic *nptII*–*dsdA* transcript in size. In addition to the monocistronic *dsdA* and di-cistronic *nptII–dsdA* transcripts, two larger RNA bands were also observed, which were most likely originated from the read-through transcription of the downstream genes (see Figure 1A) as reported in a previous work (Zhou et al., 2007). The transcript accumulation for the first cistron of the operon (*nptII* gene) was not characterized in this study, as the high kanamycin resistance of the transplastomic lines demonstrated that *nptII* mRNA was stably expressed and nptII protein accumulated to a reasonably high level (Figure 2).

### Analysis of the progeny transmission of transgenes

Reciprocal crosses between wild-type and transplastomic plants were conducted to confirm the homoplasmy of the transplastomic lines and analyze the progeny transmission of the transgenes (Figure 2). The seeds from the cross with the wild-type as the maternal parent (wt♀X Nt-pt^*dsdA*^ȷ) were spectinomycin- and kanamycin-sensitive, and the seedlings stopped growing in the medium containing either antibiotics, while the selfed transplastomic lines and the progenies from the cross with the transplastomic line as the maternal parent (Nt-pt^*dsdA*^♂X wtȷ) were resistant to both spectinomycin and kanamycin, indicating that the transgenes were stably inherited to the next generations and were maternally inherited. The lack of phenotypic segregation in T_1_ generation also suggested that the transgenes integrated into the plastid genome were indeed homoplastomic and inheritable. Taken together, the transgenes displayed the typical trait of plastid-encoded genes in tobacco (Bock, 2001; Maliga, 2004).

### Assay of D-serine resistance in transplastomic pt^dsdA^ cells and plants

**Figure 3:**
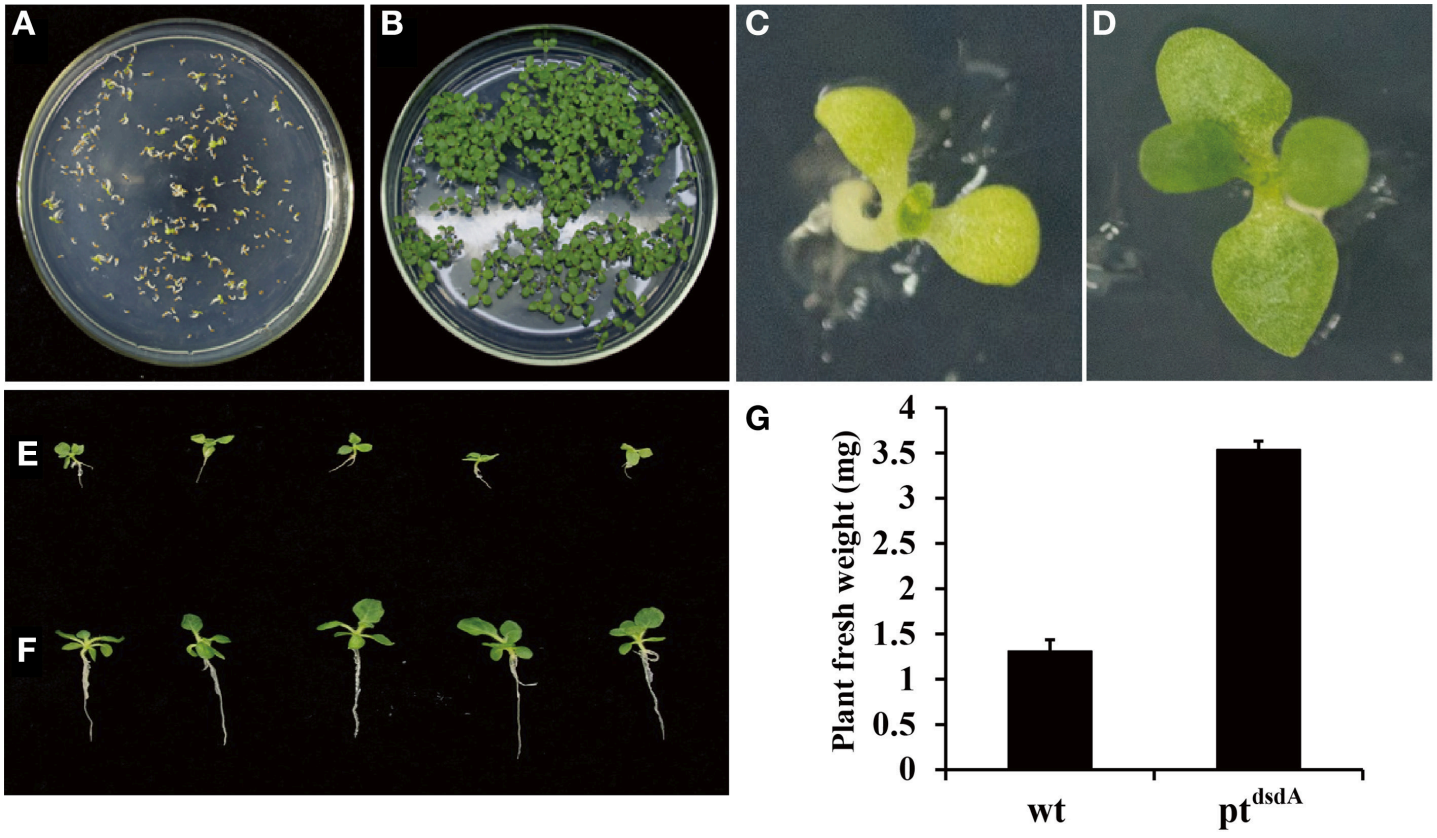
**D-serine tolerance test of transplastomic tobacco pt^dsdA^ plant**. Seedlings of wild-type and transplastomic were grown on MS medium containing 10 mM D-serine for 15 days **(A,B)** and 38 days **(C,D)** post germination. Transplastomic seedlings were cultured on medium with 10 mM D-serine **(E)** or without D-serine **(F)**. Fresh weight of aerial parts of the wild-type plant **(C)** and pt^dsdA^ plant **(D)** 38 days post germination on 10 mM D-serine medium. Error bars indicate the standard errors (SE) based on eight independent biological replicates **(G)**.

In order to analyze the effectiveness of various methods in the application of D-serine as a selective agent, three assays were performed. Firstly, the seeds from the wild-type and transplastomic pt^dsdA^ lines were grown on MS medium containing 10 mM D-serine. After 15 days of germination, the wild-type seedlings turned pale-yellow and stopped growing (Figures 3A,C), while the transplastomic seedlings developed into plants with true leaves (Figures 3B,D). Interestingly, the growth of root was inhibited compared with that of the seedlings incubated on MS without D-serine (Figures 3E,F). This phenomenon might be due to the fact that *dsdA* gene expression was driven by the chloroplast specific promoter rrn, a promoter with relatively weak transcriptional activity in roots compared with leaves (Zubko et al., 2004), which led to the decrease of D-serine tolerance in roots. In addition, the fresh weight of pt^dsdA^ plants grown on D-serine medium was much higher than that of wild-type plants (Figure 3G).

**Figure 4:**
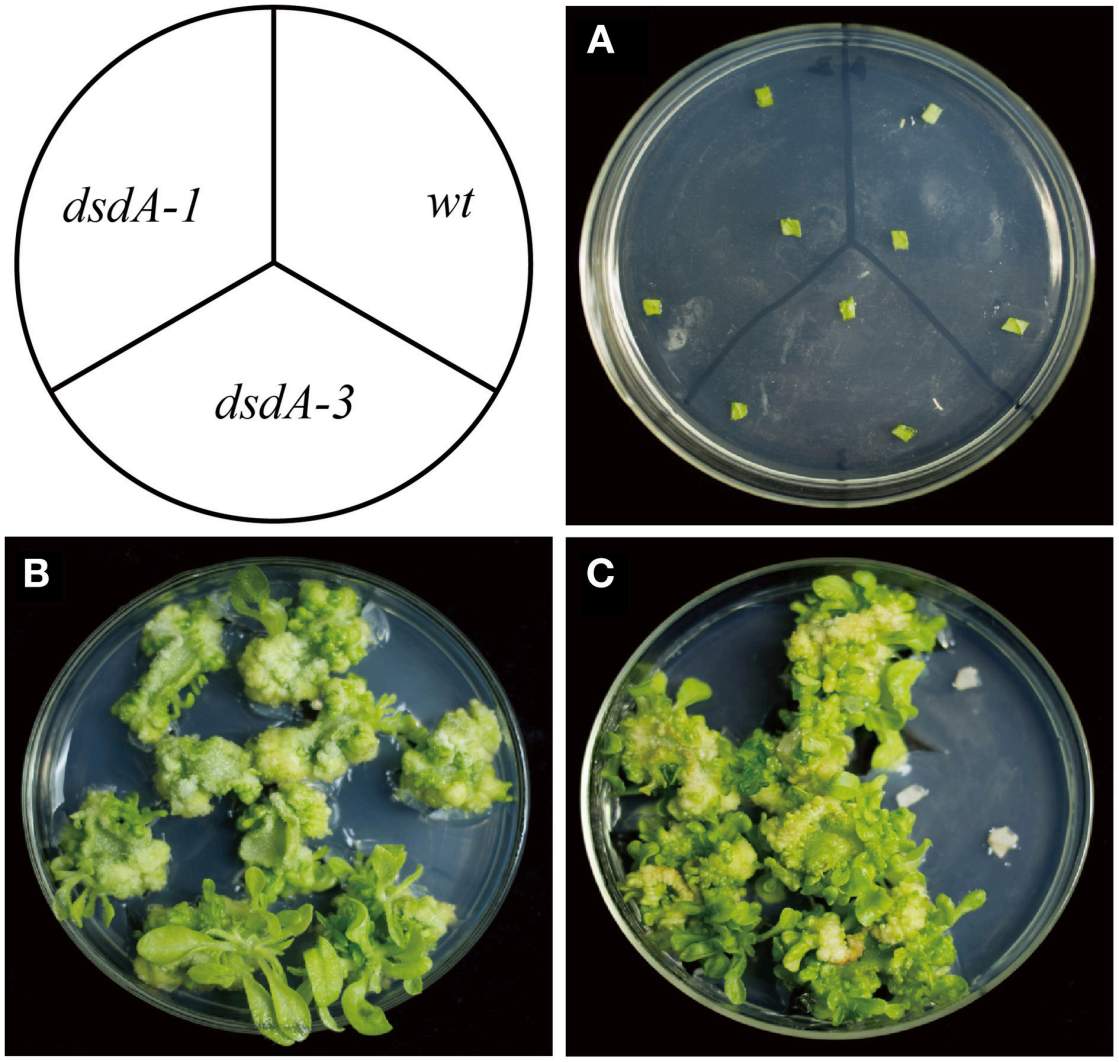
**Tolerance of transplastomic and wild-type leaves to D-serine**. Leaf explants from the transplastomic lines and the wild-type were incubated on RMOP regeneration medium on day 1 **(A)** and after 40 days without D-serine **(B)** or with 30 mM D-serine **(C)**.

Secondly, *in vitro* examination of cell division and shoot formation was performed by comparing the leaves of the wild-type and transplastomic lines cultured on RMOP plates with or without D-serine (Figure 4). When the leaves of the wild-type and transplastomic lines were cultured on RMOP plates without D-serine, no inhibition of growth was observed after 40 days of incubation (Figure 4B). On RMOP medium containing 30 mM D-serine, the leaf cells from transplastomic plants proliferated, resulting in the appearance of calluses and green shoots, while the wild-type leaf explants gradually bleached and remained aplanetic (Figure 4C). These phenotypes were obvious enough for visual separation of the DSD-expressing seedlings from the wild-type.

**Figure 5:**
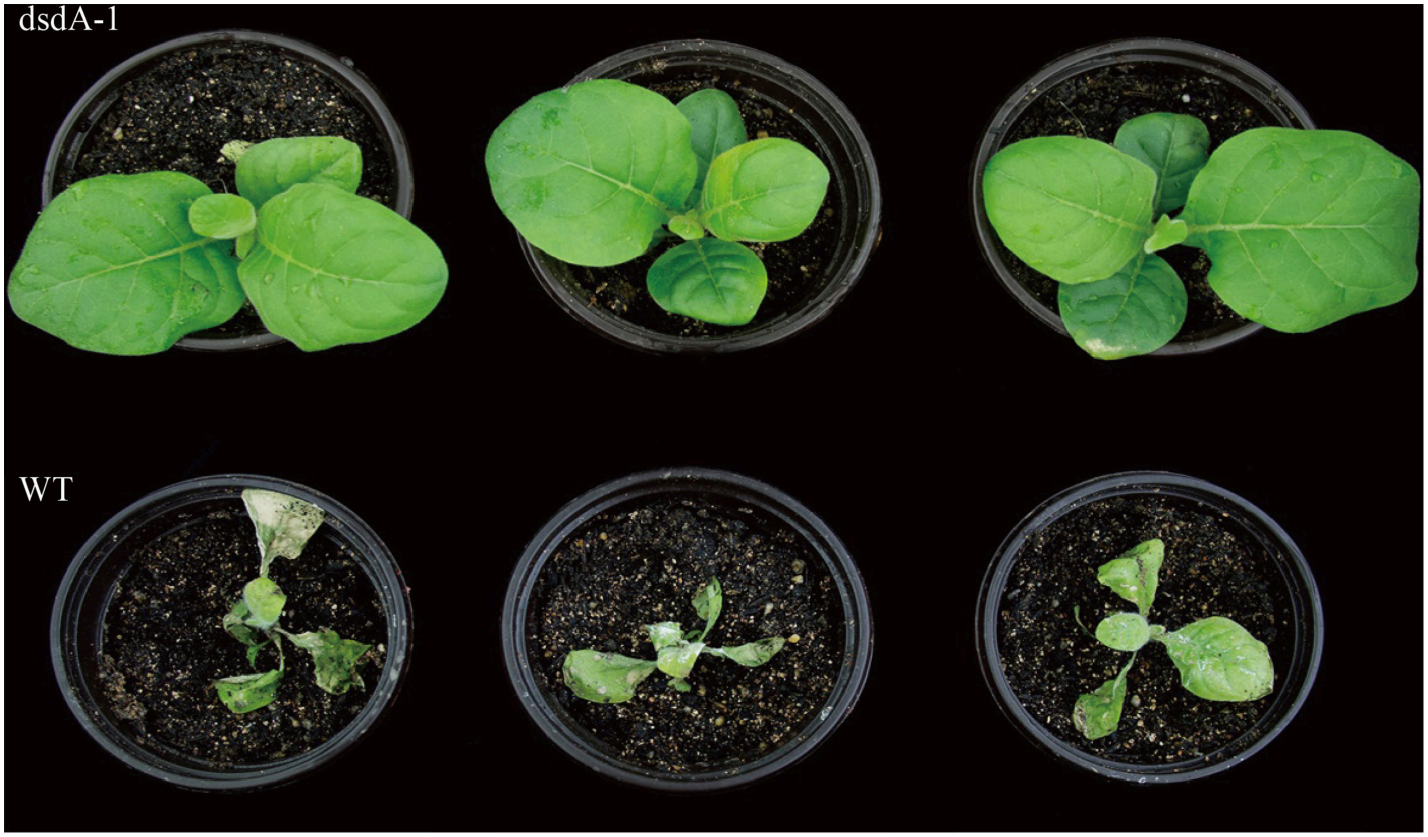
**Foliar spray testing results**. Transplastomic line **(upper)** and wild-type **(bottom)** plants grown on soil photographed after the spraying of 75 mM D-serine solution with 0.2% Tween 20 for five consecutive days. The tobaccos were grown for 5 weeks following germination before the treatment.

In the third assay, the growth of seedlings in soil was investigated. Five-week-old seedlings were sprayed with a solution of 75 mM D-serine. Transplastomic pt^dsdA^ plants were tolerant to 75 mM D-serine spraying (Figure 5, upper) and continued to grow healthily. In contrast, D-serine had adverse effect on the growth of wild-type seedlings, resulting in strong inhibition of growth and shrinking of leaves after 5 days of continuous spraying (Figure 5, bottom).

### Molecular characterization and D-serine resistance assay of transgenic plants

**Figure 6:**
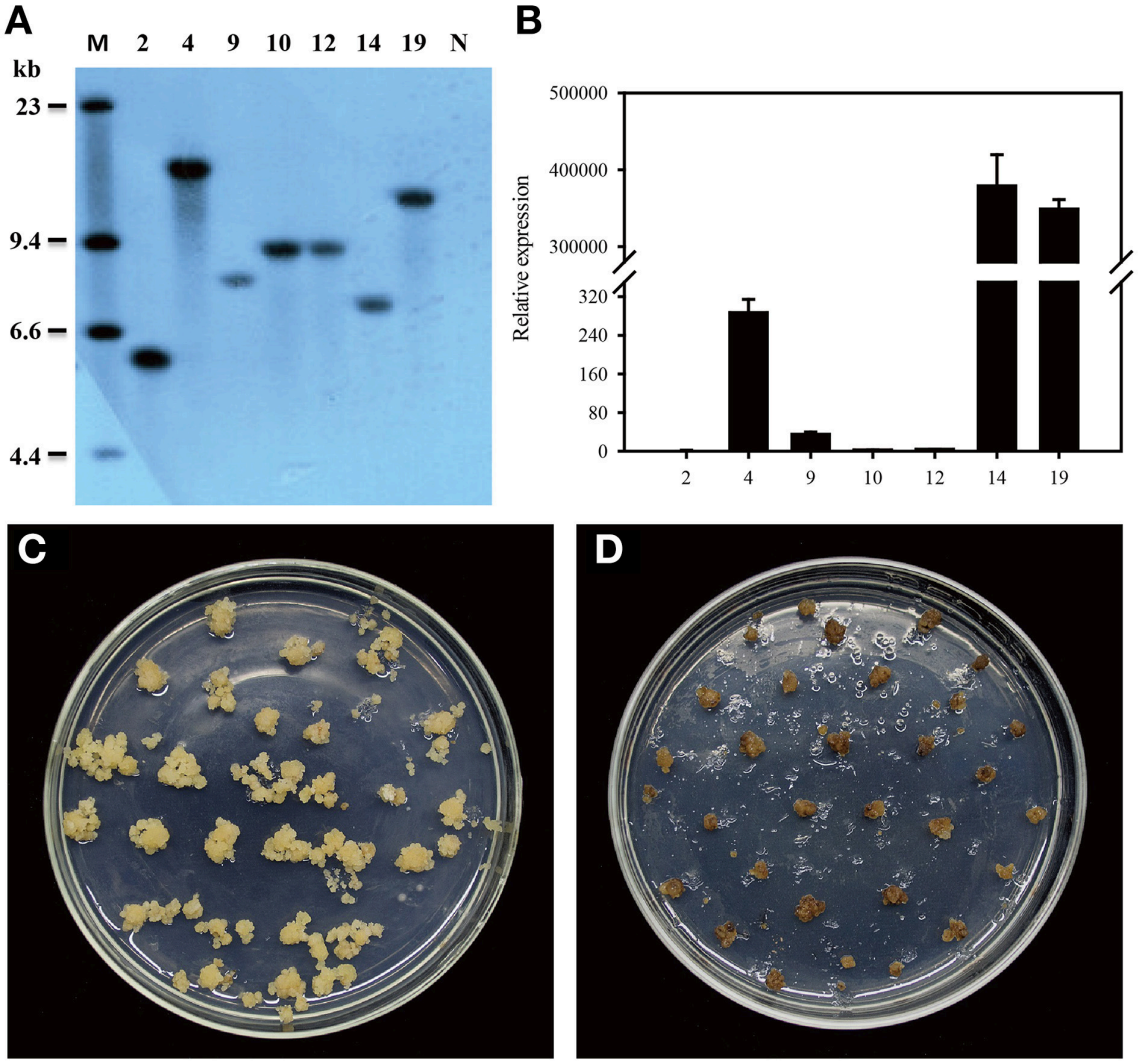
**Molecular characterization and D-serine resistance assay of transgenic plants. (A)** Southern blot analysis of genomic DNA of transgenic lines. Total genomic DNA samples were digested with HindIII and separated on a 0.8% agarose gel and hybridized with a PCR-amplified fragment of DIG-labeled hpt probe for Southern blot analysis. Marker (M); transgenic lines (2, 4, 9, 10, 12, 14, and 19); Non-transformed plant (N). **(B)** qRT-PCR was conducted to measure the relative expression of dsdA in the transgenic lines (2, 4, 9, 10, 12, 14, and 19). Resistance of the calluses from the homozygous dsdA transgenic plants with single-copy insertion **(C)** and the wild-type **(D)** placed on the selective medium containing 75 mM D-serine for 28 days.

Zhonghua 11 (*Oryza sativa* L. ssp. *japonica*) is universally considered to be a good receptor variety for transformation. Several T_1_ single-copy transgenic plants were confirmed by Southern blot (Figure 6A). To identify homozygous transgenic plants, seeds from T_1_ transgenic plants were harvested and germinated. The germination rate was recorded after 7 days of germination using wild-type plant seeds as the control. According to the Mendelian Inheritance theory, only the seeds of homozygous transgenic plants have a 100% germination rate. Homozygous *dsdA* transgenic lines were successfully isolated from all the T_1_ transgenic families. The expression level of *dsdA* in homozygous transgenic lines with single-copy insertion was examined by qRT-PCR. Three lines with high-level expression were identified among the homozygous transgenic lines (Figure 6B). In order to test the D-serine resistance of the transgenic lines, seeds from homozygous *dsdA* transgenic line with single-copy insertion and the wild-type were used for callus induction, and the calluses were respectively placed on selective medium containing 75 mM D-serine. After 28 days of selection, the calluses from the homozygous *dsdA* transgenic line showed increased resistance to D-serine and continued to regenerate (Figure 6C), whereas the wild-type calluses did not regenerate and turned necrotic (Figure 6D). These results indicate for the first time that *dsdA* gene can be potentially applied to the plastid transformation of rice.

### D-serine sensitivity of various rice species

**Figure 7:**
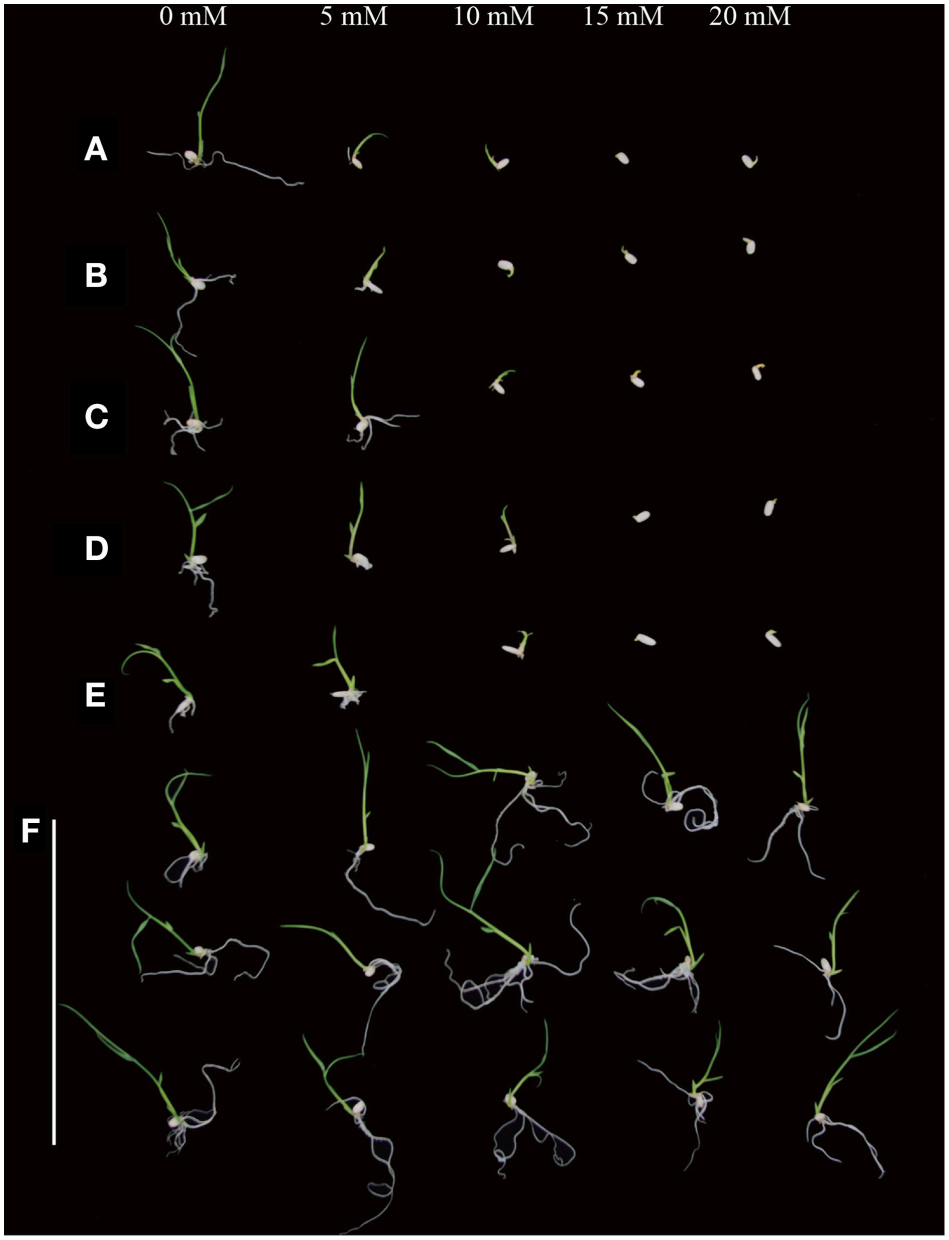
**D-serine sensitivity of various rice species**. Seeds from **(A)** Zhonghua11, **(B)** Dongjin, **(C)** Nipponbare, **(D)** Zhenshan 97 and **(E)** Minghui 63 and **(F)** the transgenic lines (4, 14, and 19, top to bottom) were surface-sterilized and germinated on fresh 1/2 × MS medium containing 0, 5, 10, 15, and 20 mM D-serine, respectively.

In order to test the sensitivity of various rice varieties to D-serine, seeds from the three *japonica* rice varieties (Zhonghua11, Dongjin, Nipponbare), two *indica* rice varieties (Minghui63 and Zhenshan 97) and the transgenic lines (4, 14, and 19) were cultured on fresh 1/2 × MS medium containing 0, 5, 10, 15, and 20 mM D-serine for 9 days. Seed germination of all the wild-type species was slightly inhibited at 5 mM D-serine, strongly inhibited at 10 mM D-serine and fully inhibited at 15 mM (Figures 7A–E). The transgenic lines could grow well even when the concentration of D-serine was 20 mM (Figure 7F). The inhibition of seed germination by D-serine indicated that D-serine could be used as selective agent in both *japonica*-type and *indica-*type rice varieties.

## Discussion

Spectinomycin has been widely used in the plastid transformation of dicots and some other species. However, as many crops are endogenously resistant to streptomycin and spectinomycin (Khan and Maliga, 1999; Li, 2013), spectinomycin cannot be used for selection in the plastid transformation of such crops. Hence, it is critical to explore new selective agents for promoting the application of plastid transformation in more species. In the present study, we explored the potential of using the selective agent D-serine for both plastid and nuclear transformation in crops. Our results show that overexpression of *dsdA* gene confers sufficient D-serine resistance to tobacco and rice. Three methods were employed to identify the effectiveness of D-serine as a selective agent in transplastomic tobacco lines. The results of all the three methods demonstrate that the resistance of the *dsdA* transplastomic line is significantly higher than that of the wild-type control, and the effective screening concentrations of D-serine are respectively 10 mM for seed germination (Figure 3), 30 mM for callus regeneration (Figure 4), and 75 mM for foliar spray (Figure 5). For the *dsdA* transgenic lines, the effective screening concentrations of D-serine are 10 mM for seed germination (Zhang et al., 2015b) and 75 mM for callus regeneration (Figure 6C). Therefore, we speculate that D-serine can be used as an effective non-antibiotic selective agent, which can efficiently select the *dsdA-*overexpressing transplastomic and transgenic crops from the wild-type plants.

In our work, D-serine resistant transformants were not obtained by direct D-serine-based selection in tobacco plastid transformation. The reason might be that the expression level of *dsdA* gene was not high enough to confer D-serine tolerance to the plants, which might be related to its expression element (this case will be discussed specifically later), or the insufficient accumulation of the target protein by the transformed plastids during the first selection stage. It has been reported that different from some selective agents such as spectinomycin, other selective agents cause cellular lethality that is related to the level of heteroplasmy (Ye et al., 2003), which might also be the case for D-serine. To overcome this difficulty, a first primary selection on spectinomycin medium can be performed, and once a sufficient proportion of D-serine resistant plastids are obtained, the calluses can be placed on the medium containing D-serine for a second selection, which is helpful for easier isolation of positive transformants from spectinomycin spontaneous mutants (Day and Goldschmidt-Clermont, 2011). On D-serine selection medium, positive transformants can continue to divide and proliferate to generate green tissues, while spectinomycin spontaneous mutants gradually turn white and then die. Transformants can also be selected with both spectinomycin and streptomycin in the second selection of plastid transformation. However, the formation of resistant shoots may be delayed under the selection with two antibiotics simultaneously (Day and Goldschmidt-Clermont, 2011). The use of D-serine as a single agent for the second selection may accelerate the formation of transplastomic callus.

In addition, with the appearance of various kinds of transgenic organisms and the increasing availability of their products, biosafety has become the focus of public concern. Particularly, antibiotic resistance marker genes have restricted the application of transgenic plants due to their potential threat to the environment and human health, and thus application of these genes in crops may be banned in the future (Kuiper et al., 2001; Miki and McHugh, 2004). Therefore, removal of the antibiotic genes from the transgenic plastids may be an effective solution to promote the commercial production of transplastomic crops. Hence, *dsdA* in our study can be a good secondary selectable marker gene after removing the *aadA* antibiotic resistance marker.

Our initial aim was to clone the synthetic *dsdA* gene into standard plastid expression cassettes with ribosomal RNA operon (P*rrn*) promoter to maximize tolerance capacity (Zhou et al., 2008; Oey et al., 2009b). However, no correct clones were obtained in *E. coli* cloning hosts, while when the *dsdA* gene was driven by the cauliflower mosaic virus (CaMV) *35S* promoter, correct clones were readily obtained (Erikson et al., 2004; Zhang et al., 2015b). We therefore, suspected that high accumulation of this gene under the control of prokaryotic-type plastid expression signals might be lethal to bacteria. Thus, we chose to employ the previously described pZF75-based plastid transformation vector (Zhou et al., 2007) and put *dsdA* gene as the second cistron in the expression operon. As reported previously by Oey et al. (2009b), the terminator from the upstream genes can block the transcription of the downstream genes in *E. coli*, but not in plant plastid system. Our results showed that the expression level of *dsdA* in plant leaves was high and the transformants showed strong resistance to D-serine (up to 75 mM), indicating that the *rrn* promoter facilitates strong and constitutive expression of this gene in plastid. However, it is clearly demonstrated that the use of *rrn* promoter cannot provide sufficient tolerance to 10 mM D-serine in root (Figures 3E,F). Therefore, to achieve efficient and effective selection in plastid transformation, it is necessary to apply an effective *cis*-element to promote the gene expression in non-green plastids, such as P_*ClpP*_ (Zhang et al., 2012), P_*accD*_ (Caroca et al., 2013), and more diversities should be considered.

Our results indicate for the first time that *dsdA* gene can be potentially applied for selecting and screening both nuclear and plastid transformants in rice. The inhibition of seed germination by D-serine suggests that D-serine is toxic to both *japonica*-type and *indica*-type rice varieties (Figure 7), indicating that D-serine may be commonly used as a non-antibiotic selective agent in different rice varieties. As for *dsdA*, it catalyzes the deamination of the D-serine to pyruvate, ammonium and water, which participate in the metabolism of plants. No direct interference with endogenous amino acid metabolism was found when *dsdA* was expressed in *A. thaliana* (Erikson et al., 2005). The expression of *dsdA* in maize plants had no adverse effects on agronomics, yield, or grain composition (Lai et al., 2011). In our study, no retarded growth in transgenic *dsdA* rice lines was observed (Figure S1A), and the statistical analysis of chlorophyll content, plant growth and root development showed no significant differences between the *dsdA* transgenic lines and the wild-type control (Figures S1B–D). Overall, our research proves the feasibility of using the *dsdA* gene as a selective marker in both plastid and nuclear transformation systems, and provides an effective candidate selective gene to promote the application of plastid transformation in monocotyledons.

## Author contributions

YML, YJL, and FZ conceived and designed the experiments. YML, ZH, HL, SL, and JZ conducted the experiments. YML, RW, and FZ analyzed the results. YML, RW, and FZ wrote the paper. YML, RW, YJL, and FZ revised the paper. All authors reviewed the manuscript.

### Conflict of interest statement

The authors declare that the research was conducted in the absence of any commercial or financial relationships that could be construed as a potential conflict of interest.

## Supplementary material

### Materials and methods

Leaves (approximately 0.02 g fresh weight) were collected and soaked in 1.5 mL of acetone (4.5): ethanol (4.5): H_2_O (1) miscible liquids at 4°C for 24 h in dark. The supernatant was analyzed with a DU640 UV/Vis Spectrophotometer at 663 and 645 nm, respectively. Measurement of chlorophyll a (Chla) and chlorophyll b (Chlb) was conducted following the method of Arnon (1949).

Figure S1 **The phenotypic and statistical analysis of all the lines at vegetative stage. (A)** The *dsdA* transgenic lines showed no retarded growth compared with the wild-type (ZH11) control. **(B)** Analysis of chlorophyll content in *dsdA* transgenic lines. The contents of Chla and Chlb in *dsdA* transgenic lines are not significantly different from those in the wild-type control lines. Error bars indicate the standard errors (SE) based on three biological replicates. **(C)** Analysis of plant growth. The plant height of all the lines at vegetative stage was measured. Error bars indicate SE based on six independent biological replicates. **(D)** Analysis of root development. The root length of all the lines at seedling stage (3 weeks) was measured. The vertical bars represent SE for four independent biological replicates.

## References

[B1] Arnon D. I. (1949). Copper enzymes in isolated chloroplasts. polyphenoloxidase in *Beta vulgaris*. Plant Physiol. 24, 1–15. 10.1104/pp.24.1.1 16654194 PMC437905

[B2] Barone P. Zhang X. H. Widholm J. M. (2009). Tobacco plastid transformation using the feedback-insensitive anthranilate synthase [alpha]-subunit of tobacco (ASA2) as a new selectable marker. J. Exp. Bot. 60, 3195–3202. 10.1093/jxb/erp160 19553372 PMC2718221

[B3] Bock R. (2001). Transgenic plastids in basic research and plant biotechnology. J. Mol. Biol. 312, 425–438. 10.1006/jmbi.2001.4960 11563907

[B4] Bock R. (2014). Genetic engineering of the chloroplast: novel tools and new applications. Curr. Opin. Biotechnol. 26, 7–13. 10.1016/j.copbio.2013.06.004 24679252

[B5] Caroca R. Howell K. A. Hasse C. Ruf S. Bock R. (2013). Design of chimeric expression elements that confer high-level gene activity in chromoplasts. Plant J. 73, 368–379. 10.1111/tpj.12031 23004223

[B6] Carrer H. Hockenberry T. N. Svab Z. Maliga P. (1993). Kanamycin resistance as a selectable marker for plastid transformation in tobacco. Mol. Gen. Genet. 241, 49–56. 10.1007/BF00280200 8232211

[B7] Chen H. Tang W. Xu C. Li X. Lin Y. Zhang Q. (2005). Transgenic indica rice plants harboring a synthetic cry2A^*^ gene of *Bacillus thuringiensis* exhibit enhanced resistance against lepidopteran rice pests. Theor. Appl. Genet. 111, 1330–1337. 10.1007/s00122-005-0062-8 16187120

[B8] Clarke J. L. Waheed M. T. Lossl A. G. Martinussen I. Daniell H. (2013). How can plant genetic engineering contribute to cost-effective fish vaccine development for promoting sustainable aquaculture?. Plant Mol. Biol. 83, 33–40. 10.1007/s11103-013-0081-9 23729352 PMC3755229

[B9] Craig W. Lenzi P. Scotti N. De Palma M. Saggese P. Carbone V.. (2008). Transplastomic tobacco plants expressing a fatty acid desaturase gene exhibit altered fatty acid profiles and improved cold tolerance. Transgene Res. 17, 769–782. 10.1007/s11248-008-9164-9 18214708

[B10] Daniell H. Dhingra A. (2002). Multigene engineering: dawn of an exciting new era in biotechnology. Curr. Opin. Biotechnol. 13, 136–141. 10.1016/S0958-1669(02)00297-5 11950565 PMC3481857

[B11] D'Aniello A. D'onofrio G. Pischetola M. D'aniello G. Vetere A. Petrucelli L.. (1993). Biological role of D-amino acid oxidase and D-aspartate oxidase. effects of D-amino acids. J. Biol. Chem. 268, 26941–26949. 7903300

[B12] Day A. Goldschmidt-Clermont M. (2011). The chloroplast transformation toolbox: selectable markers and marker removal. Plant Biotechnol. J. 9, 540–553. 10.1111/j.1467-7652.2011.00604.x 21426476

[B13] De Miranda J. Panizzutti R. Foltyn V.N. Wolosker H. (2002). Cofactors of serine racemase that physiologically stimulate the synthesis of the N-methyl-Daspartate (NMDA) receptor coagonist D-serine. Proc. Natl. Acad. Sci. U.S.A. 99, 14542–14547. 10.1073/pnas.222421299 12393813 PMC137919

[B14] Dhingra A. Portis A. R. Jr. Daniell H. (2004). Enhanced translation of a chloroplast-expressed *RbcS* gene restores small subunit levels and photosynthesis in nuclear *RbcS* antisense plants. Proc. Natl. Acad. Sci. U.S.A. 101, 6315−6320. 10.1073/pnas.0400981101 15067115 PMC395966

[B15] Doyle J. J. Doyle J. L. (1990). Isolation of plant DNA from fresh tissue. Focus 12, 13–15.

[B16] Erikson O. Hertzberg M. Näsholm T. (2004). A conditional marker gene allowing both positive and negative selection in plants. Nat. Biotechnol. 22, 455–458. 10.1038/nbt946 15085802

[B17] Erikson O. Hertzberg M. Näsholm T. (2005). The *dsdA* gene from *Escherichia coli* provides a novel selectable marker for plant transformation. Plant Mol. Biol. 57, 425–433. 10.1007/s11103-004-7902-9 15830131

[B18] Federiuk C. S. Bayer R. Shafer J. A. (1983). Characterization of the catalytic pathway for D-serine dehydratase. *J. Biol*. Chem. 258, 5379–5385. 6406501

[B19] Friedman M. (1999). Chemistry, nutrition, and microbiology of D-amino acids. J. Agric. Food Chem. 47, 3457–3479. 10.1021/jf990080u 10552672

[B20] Gisby M. F. Mudd E. A. Day A. (2012). Growth of transplastomic cells expressing D-amino acid oxidase in chloroplasts is tolerant to D-alanine and inhibited by D-valine. Plant Physiol. 160, 2219–2226. 10.1104/pp.112.204107 23085840 PMC3510142

[B21] Gorantala J. Grover S. Rahi A. Chaudhary P. Rajwanshi R. Sarin N. B.. (2014). Generation of protective immune response against anthrax by oral immunization with protective antigen plant-based vaccine. J. Biotechnol. 176, 1–10. 10.1016/j.jbiotec.2014.01.033 24548460

[B22] Hassan S. W. Waheed M. T. Müller M. Clarke J. L. Shinwari Z. K. Lössl A. G. (2014). Expression of HPV-16 L1 capsomeres with glutathione-S-transferase as a fusion protein in tobacco plastids: an approach for a capsomere-based HPV vaccine. Hum. Vaccin. Immunother. 10, 2975–2982. 10.4161/21645515.2014.970973 25483463 PMC5443053

[B23] Hiei Y. Ohta S. Komari T. Kumashiro T. (1994). Effiient transformation of rice (*Oryza sativa* L.) mediated by Agrobacterium and sequence analysis of the boundaries of the T-DNA. Plant J. 6, 271–282. 10.1046/j.1365-313X.1994.6020271.x 7920717

[B24] Hou B. K. Zhou Y. H. Wan L. H. Zhang Z. L. Shen G. F. Chen Z. H.. (2003). Chloroplast transformation in oilseed rape. Transgene Res. 12, 111–114. 10.1023/A:1022180315462 12650529

[B25] Kang T. J. Seo J. E. Loc N. H. Yang M. S. (2003). Herbicide resistance of tobacco chloroplasts expressing the bar gene. Mol. Cell. 16, 60–66. 14503846

[B26] Khan M. S. Maliga P. (1999). Fluorescent antibiotic resistance marker for tracking plastid transformation in higher plants. Nat. Biotechnol. 17, 910–915. 10.1038/12907 10471936

[B27] Kolotilin I. Kaldis A. Devriendt B. Joensuu J. Cox E. Menassa R. (2012). Production of a subunit vaccine candidate against porcine post-weaning diarrhea in high-biomass transplastomic tobacco. PLoS ONE 7:e42405. 10.1371/journal.pone.0042405 22879967 PMC3411772

[B28] Koya V. Moayeri M. Leppla S. H. Daniell H. (2005). Plant-based vaccine: mice immunized with chloroplast-derived anthrax protective antigen survive anthrax lethal toxin challenge. Infect. Immun. 73, 8266–8274. 10.1128/IAI.73.12.8266-8274.2005 16299323 PMC1307059

[B29] Krishnamoorthy G. Sadulla S. Sehgal P. K. Mandal A. B. (2012). Green chemistry approaches to leather tanning process for making chrome-free leather by unnatural amino acids. J. Hazard. Mater. 216, 173–182. 10.1016/j.jhazmat.2012.02.046 22421341

[B30] Krug A. W. Völker K. Dantzler W. H. Silbernagl S. (2007). Why is D-serine nephrotoxic and α-aminoisobutyric acid protective? Am. J. Physiol. Renal Physiol. 293, F382–F390. 10.1152/ajprenal.00441.2006 17429029

[B31] Kuiper H. A. Kleter G. A. Noteborn H. P. J. M. Kok E. J. (2001). Assessment of the food safety issues related to genetically modified foods. Plant J. 27, 503–528. 10.1046/j.1365-313X.2001.01119.x 11576435

[B32] Kumar S. Dhingra A. Daniell H. (2004). Plastid-expressed betaine aldehyde dehydrogenase gene in carrot cultured cells, roots, and leaves confers enhanced salt tolerance. Plant Physiol. 136, 2843–2854. 10.1104/pp.104.045187 15347789 PMC523346

[B33] Kuroda H. Maliga P. (2001). Complementarity of the 16S rRNA penultimate stem with sequences downstream of the AUG destabilizes the plastid mRNAs. Nucleic Acids Res. 29, 970–975. 10.1093/nar/29.4.970 11160930 PMC29611

[B34] Kuru E. Hughes H. V. Brown P. J. Hall E. Tekkam S. Cava F.. (2012). *In situ* probing of newly synthesized peptidoglycan in live bacteria with fluorescent D-amino acids. Angew. Chem. 51, 12519−12523. 10.1002/anie.201206749 23055266 PMC3589519

[B35] Lai F. M. Privalle L. Mei K. Ghoshal D. Shen Y. Klucinec J. (2011). Evaluation of the E. coli D-serine ammonia lyase gene *(Ec. dsdA)* for use as a selectable marker in maize transformation. In vitro Cell. Dev. Biol. Plant. 47, 467–479. 10.1007/s11627-011-9351-x

[B36] Lee S. B. Kwon H. B. Kwon S. J. Park S. C. Jeong M. J. Han S. E. (2003). Accumulation of a trehalose within transgenic chloroplasts confers drought tolerance. Mol. Breed. 11, 1–13. 10.1023/A:1022100404542

[B37] Lee S. M. Kang K. Chung H. Yoo S. H. Xu X. M. Lee S. B.. (2006). Plastid transformation in the monocotyledonous cereal crop, rice (*Oryza sativa*) and transmission of transgenes to their progeny. Mol. Cells 21, 401–410. 16819304 PMC3481850

[B38] Li D. (2013). Establishment of the Chloroplast Genetic Transformation System in Rice by using Hygromycin B as Selection Pressure. Ph.D. Dissertation, Central South University.

[B39] Li W. Ruf S. Bock R. (2011). Chloramphenicol acetyltransferase as selectable marker for plastid transformation. Plant Mol. Biol. 76, 443–451. 10.1007/s11103-010-9678-4 20721602

[B40] Li X. Li S. Lang Z. Zhang J. Zhu L. Huang D. (2013). Chloroplast-targeted expression of the codon-optimized truncated cry1Ah gene in transgenic tobacco confers a high level of protection against insects. Plant Cell Rep. 32, 1299–1308. 10.1007/s00299-013-1444-z 23620344

[B41] Liu C. W. Lin C. C. Yiu J. C. Chen J. J. W. Tseng M. J. (2008). Expression of a *Bacillus thuringiensis* toxin (*cry1Ab*) gene in cabbage *(Brassica oleracea L. var. capitata L.)* chloroplasts confers high insecticidal efficacy against *Plutella xylostella*. Theor. Appl. Genet. 117, 75–88. 10.1007/s00122-008-0754-y 18415072

[B42] Livak K. J. Schmittgen T. D. (2001). Analysis of relative gene expression data using real-time quantitative PCR and the 2^−ΔΔCT^ method. Methods 25, 402–408. 10.1006/meth.2001.1262 11846609

[B43] Lu Y. Rijzaani H. Karcher D. Ruf S. Bock R. (2013). Efficient metabolic pathway engineering in transgenic tobacco and tomato plastids with synthetic multigene operons. Proc. Natl. Acad. Sci. U.S.A. 110, E623–E632. 10.1073/pnas.1216898110 23382222 PMC3581966

[B44] Maliga P. (2004). Plastid transformation in higher plants. Annu. Rev. Plant Biol. 55, 289–313. 10.1146/annurev.arplant.55.031903.141633 15377222

[B45] Miki B. McHugh S. (2004). Selectable marker genes in transgenic plants: applications, alternatives and biosafety. J. Biotechnol. 107, 193–232. 10.1016/j.jbiotec.2003.10.011 14736458

[B46] Murashige T. Skoog F. (1962). A revised medium for rapid growth and bio assays with tobacco tissue culture. Physiol. Plant. 15, 473–497. 10.1111/j.1399-3054.1962.tb08052.x

[B47] Murray M. G. Thompson W. F. (1980). Rapid isolation of high molecular weight plant DNA. Nucleic Acids Res. 8, 4321–4325. 10.1093/nar/8.19.4321 7433111 PMC324241

[B48] Oey M. Lohse M. Kreikemeyer B. Bock R. (2009a). Exhaustion of the chloroplast protein synthesis capacity by massive expression of a highly stable protein antibiotic. Plant J. 57, 436–445. 10.1111/j.1365-313X.2008.03702.x 18939966

[B49] Oey M. Lohse M. Scharff L. B. Kreikemeyer B. Bock R. (2009b). Plastid production of protein antibiotics against pneumonia via a new strategy for high-level expression of antimicrobial proteins. Proc. Natl. Acad. Sci. U.S.A. 106, 6579–6584. 10.1073/pnas.0813146106 19332784 PMC2662961

[B50] Ruf S. Hermann M. Berger I. J. Carrer H. Bock R. (2001). Stable genetic transformation of tomato plastids and expression of a foreign protein in fruit. Nat. Biotechnol. 19, 870–875. 10.1038/nbt0901-870 11533648

[B51] Ruf S. Karcher D. Bock R. (2007). Determining the transgene containment level provided by chloroplast transformation. Proc. Natl. Acad. Sci. U.S.A. 104, 6998–7002. 10.1073/pnas.0700008104 17420459 PMC1849964

[B52] Ruhlman T. Verma D. Samson N. Daniell H. (2010). The role of heterologous chloroplast sequence elements in transgene integration and expression. Plant Physiol. 152, 2088–2104. 10.1104/pp.109.152017 20130101 PMC2850035

[B53] Schneider A. Stelljes C. Adams C. Kirchner S. Burkhard G. Jarzombski S.. (2015). Low frequency paternal transmission of plastid genes in *Brassicaceae*. Transgenic Res. 24, 267–277. 10.1007/s11248-014-9842-8 25343875

[B54] Scotti N. Alagna F. Ferraiolo E. Formisano G. Sannino L. Buonaguro L.. (2009). High-level expression of the HIV-1 Pr55gag polyprotein in transgenic tobacco chloroplasts. Planta 229, 1109–1122. 10.1007/s00425-009-0898-2 19234717

[B55] Soria-Guerra R. E. Alpuche-Solís A. G. Rosales-Mendoza S. Moreno-Fierros L. Bendik E. M. Martínez-González L.. (2009). Expression of a multi-epitope DPT fusion protein in transplastomic tobacco plants retains both antigenicity and immunogenicity of all three components of the functional oligomer. Planta 229, 1293–1302. 10.1007/s00425-009-0918-2 19306020 PMC7087907

[B56] Staub J. M. Maliga P. (1994). Translation of the *psbA* mRNA is regulated by light via the 5'-untranslated region in tobacco plastids. Plant J. 6, 547–553. 10.1046/j.1365-313X.1994.6040547.x 7987413

[B57] Svab Z. Hajdukiewicz P. Maliga P. (1990a). Stable transformation of plastids in higher plants. Proc. Natl. Acad. Sci. U.S.A. 87, 8526–8530. 10.1073/pnas.87.21.8526 11607112 PMC54989

[B58] Svab Z. Harper E. C. Jones J. D. Maliga P. (1990b). Aminoglycoside-3″-adenyltransferase confers resistance to spectinomycin and streptomycin in *Nicotiana tabacum*. Plant Mol. Biol. 14, 197–205. 10.1007/BF00018560 1966273

[B59] Svab Z. Maliga P. (1993). High-frequency plastid transformation in tobacco by selection for a chimeric aadA gene. Proc. Natl. Acad. Sci. U.S.A. 90, 913–917. 10.1073/pnas.90.3.913 8381537 PMC45780

[B60] Tregoning J. S. Nixon P. Kuroda H. Svab Z. Clare S. Bowe F.. (2003). Expression of tetanus toxin fragment C in tobacco chloroplasts. Nucleic Acids Res. 31, 1174–1179. 10.1093/nar/gkg221 12582236 PMC150239

[B61] Tsai G. Yang P. Chung L. C. Lange N. Coyle J. T. (1998). D-serine added to antipsychotics for the treatment of schizophrenia. Biol. Psychiatry 44, 1081–1089. 10.1016/S0006-3223(98)00279-0 9836012

[B62] Valdovinos J. G. Muir R. M. (1965). Effects of D and L amino acids on foliar abscission. Plant Physiol. 40, 335–340. 10.1104/pp.40.2.335 16656091 PMC550290

[B63] Wang R. Lu L. Pan X. Hu Z. Ling F. Yan Y.. (2015). Functional analysis of *OsPGIP1* in rice sheath blight resistance. Plant Mol. Biol. 87, 181–191. 10.1007/s11103-014-0269-7 25488398

[B64] Wurbs D. Ruf S. Bock R. (2007). Contained metabolic engineering in tomatoes by expression of carotenoid biosynthesis genes from the plastid genome. Plant J. 49, 276–288. 10.1111/j.1365-313X.2006.02960.x 17241450

[B65] Ye G. N. Colburn S. M. Xu C. W. Hajdukiewicz P. T. J. Staub J. M. (2003). Persistence of unselected transgenic DNA during a plastid transformation and segregation approach to herbicide resistance. Plant Physiol. 133, 402–410. 10.1104/pp.103.021949 12970505 PMC196616

[B66] Ye G. N. Hajdukiewicz P. T. Broyles D. Rodriguez D. Xu C. W. Nehra N.. (2001). Plastid-expressed 5-enolpyruvylshikimate-3-phosphate synthase genes provide high level glyphosate tolerance in tobacco. Plant J. 25, 261–270. 10.1046/j.1365-313x.2001.00958.x 11208018

[B67] Zhang J. J. Lin Y. J. Zhou F. (2015b). Identification and application of new selectable marker genes for rice nuclear transformation. J. Huazhong Agric. Univ. 34, 1–8

[B68] Zhang J. Khan S. A. Hasse C. Ruf S. Heckel D. G. Bock R. (2015a). Full crop protection from an insect pest by expression of long double-stranded RNAs in plastids. Science 347, 991–994. 10.1126/science.1261680 25722411

[B69] Zhang J. Ruf S. Hasse C. Childs L. Scharff L. B. Bock R. (2012). Identification of *cis*-elements conferring high levels of gene expression in non-green plastids. Plant J. 72, 115–128. 10.1111/j.1365-313X.2012.05065.x 22639905

[B70] Zhou F. Badillo-Corona J. A. Karcher D. Gonzalez-Rabade N. Piepenburg K. Borchers A. M. (2008). High-level expression of HIV antigens from the tobacco and tomato plastid genomes. Plant Biotechnol J. 6, 897–913. 10.1111/j.1467-7652.2008.00356.x 19548344

[B71] Zhou F. Karcher D. Bock R. (2007). Identification of a plastid intercistronic expression element (IEE) facilitating the expression of stable translatable monocistronic mRNAs from operons. Plant J. 52, 961–972. 10.1111/j.1365-313X.2007.03261.x 17825052 PMC2230500

[B72] Zubko M. K. Zubko E. I. Van Zuilen K. Meyer P. Day A. (2004). Stable transformation of petunia plastids. Transgenic Res. 13, 523–530. 10.1007/s11248-004-2374-x 15672833

