# Supplementary figures and images for: Expression of a Codon-Optimized dsdA Gene in Tobacco Plastids and Rice Nucleus Confers D-Serine Tolerance
Source: Front Plant Sci. 2016 May 12;7:640. doi: 10.3389/fpls.2016.00640 (PMC4863892; doi:10.3389/fpls.2016.00640)

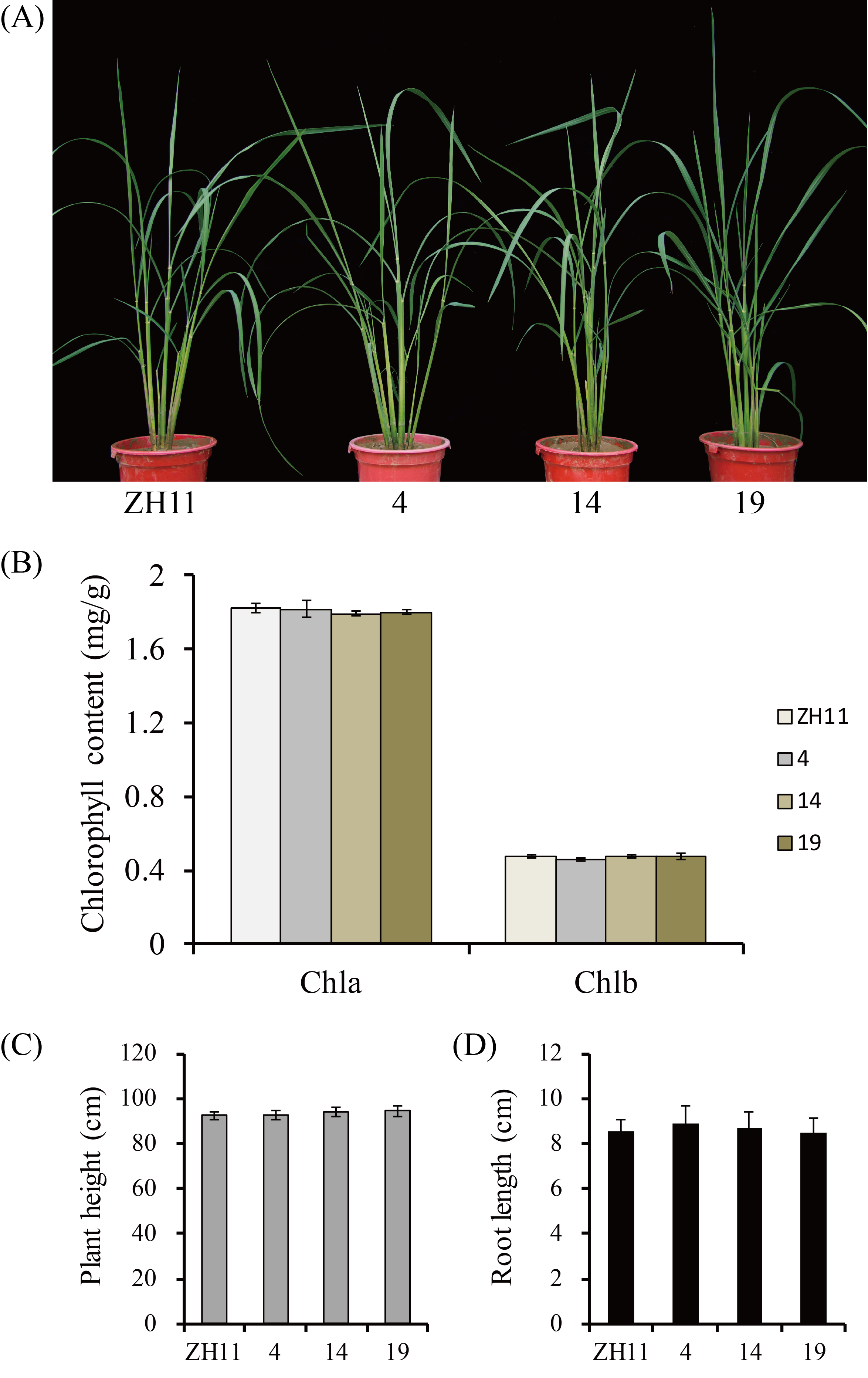

Supplement: Figure S1 — The phenotypic and statistical analysis of all the lines at vegetative stage. (A) The dsdA transgenic lines showed no retarded growth compared with the wild-type (ZH11) control. (B) Analysis of chlorophyll content in dsdA transgenic lines. The contents of Chla and Chlb in dsdA transgenic lines are not significantly different from those in the wild-type control lines. Error bars indicate the standard errors (SE) based on three biological replicates. (C) Analysis of plant growth. The plant height of all the lines at vegetative stage was measured. Error bars indicate SE based on six independent biological replicates. (D) Analysis of root development. The root length of all the lines at seedling stage (3 weeks) was measured. The vertical bars represent SE for four independent biological replicates. [file Image1.JPEG]
