# Supplementary material for: Expression of a Codon-Optimized dsdA Gene in Tobacco Plastids and Rice Nucleus Confers D-Serine Tolerance
Source: Front Plant Sci. 2016 May 12;7:640. doi: 10.3389/fpls.2016.00640 (PMC4863892; doi:10.3389/fpls.2016.00640)
Supplement: Supplementary file 2 [file DataSheet1.DOCX]

**File 1.** The codon-optimized *dsdA* sequence. CATATGGAAAATGCTAAAATGAACTCTTTAATTGCTCAATATCCTTTAGTTAAAGATTTAGTTGCTTTAAAGGAAACTACTTGGTTTAATCCTGGAACTACTTCTTTAGCTGAAGGATTACCTTATGTTGGATTAACTGAACAAGATGTTCAAGATGCTCATGCTAGATTATCCAGATTTGCTCCTTATTTAGCTAAAGCATTTCCTGAAACTGCTGCTACTGGAGGAATTATTGAATCTGAATTAGTTGCTATTCCTGCTATGCAGAAAAGATTAGAGAAGGAATATCAACAACCTATTTCTGGACAATTATTATTAAAGAAAGATTCTCATTTACCTATTTCTGGATCTATTAAAGCTAGAGGAGGAATTTATGAAGTATTAGCTCATGCTGAAAAATTAGCTTTAGAAGCTGGATTATTAACTTTAGATGATGATTATTCTAAATTATTATCTCCTGAATTTAAACAATTCTTTTCTCAATATTCTATTGCTGTTGGATCTACTGGAAATTTAGGATTATCTATTGGAATTATGTCTGCTAGAATTGGATTTAAAGTTACTGTTCACATGTCTGCTGATGCTAGAGCTTGGAAGAAAGCTAAATTACGTTCTCATGGAGTTACTGTTGTTGAATATGAACAAGATTATGGAGTTGCTGTTGAAGAAGGAAGAAAAGCTGCTCAATCTGATCCTAATTGTTTCTTTATTGATGATGAAAATTCCAGAACTTTATTTCTTGGATATTCTGTTGCTGGACAAAGATTAAAAGCTCAATTTGCTCAACAAGGAAGAATTGTTGATGCTGATAATCCTTTATTCGTTTATTTACCTTGTGGAGTTGGAGGAGGACCTGGAGGAGTTGCATTTGGATTAAAATTAGCATTTGGAGATCATGTTCATTGCTTCTTTGCTGAACCTACTCATTCTCCTTGTATGTTATTAGGAGTTCATACTGGATTACATGATCAAATATCTGTTCAAGATATTGGAATTGATAATTTAACTGCTGCTGATGGATTAGCTGTTGGAAGAGCTTCTGGATTTGTTGGAAGAGCTATGGAAAGATTATTAGATGGATTCTATACTTTATCTGATCAAACTATGTATGATATGTTAGGATGGTTAGCTCAAGAAGAAGGAATTAGATTAGAACCTTCTGCTTTAGCTGGAATGGCTGGACCTCAAAGAGTTTGTGCTTCTGTTTCTTATCAACAAATGCATGGATTTTCTGCTGAACAATTAAGAAATACTACTCATTTAGTTTGGGCTACTGGAGGAGGAATGGTTCCTGAAGAAGAAATGAATCAATATTTAGCTAAAGGAAGATAATTCTAGA
